# Amblyopia from Errors of Refraction

**Published:** 1891-10-31

**Authors:** 


### OPHTHALMOLOGY.

### AMBLYOPIA FROM ERRORS OF REFRACTION.

Amblyopia is a defect of vision not amounting to blindness, a diminution of visual acuity, a want of the retina to act to the usual stimulus. It arises from many diseases, but it is often the result of errors of refraction without any pathological condition of the refractive media.

In testing the sight by aid of Snellen's types, or any tests for near and far vision, we may find a patient with an excellent range of vision, who, with one eye covered, may possess the normal range of sight, and yet with the other have a very limited range. This is often met with in high hypermetropia, more especially when the eyes have an unequal amount, in hypermetropic astigmatism, when one eye is hypermetropic and the other eye myopic, or in myopia of different degree in either sex. Amblyopia arising from errors of refraction may be described as unsymmetrical amblyopia, though there is one form, due to overwork, of hyperopic eyes at near points, producing spasm of the ciliary muscles, and consequent failure of accommodation, in which the amblyopia is symmetrical. The amblyopia from errors of refraction shows no ophthalmoscopic changes, the media are found clear, and the fundus normal.

How does the amblyopia come about? When the subjects of these errors of refraction commence to use the eyes for near objects, as in learning to read, write, sew, &c., he finds that a great effort is necessary to bring the two eyes, different in refractive degree, to a symmetrical focus, and instinctively learns to suppress the image in the most affected eye.

In unequal hypermetropia the effort to focus an object clearly, ceases when one eye, that with the less hypermetropia, is satisfied, and the image is thus never clearly defined in the other.

In unequal myopia, objects held at the focus for one eye cannot be seen clearly in the other eye, and if, as often happens, the patient always uses the same eye, the other never receives sufficient stimulus, and becomes amblyopic.

Again, the act of convergence to focus near objects may overtax the internal rectus muscle of one eyeball, and spasm occur, causing internal strabismus. The patient then focuses objects on the two retinæ at points which do not correspond, and therefore gets two retinal images, one bright, clear, and sharp in outline, the other looking like its ghost, so to speak. He then soon learns to suppress the blurred or ghostly image, and the eye from disuse becomes amblyopic. The acuity of vision of the retina for defining objects and answering to the stimulus of light diminishes, just as the muscles of an athlete diminish when he gives up his training and athletic exercise. The retina still is unconsciously stimulated by light, and the images formed on it by surrounding objects, although nothing is focussed clearly by it, and is thus prevented from entirely losing its power, just as the athlete's muscles receive sufficient stimulus from his ordinary exercise and employment of them in performing the necessary duties of daily existence to prevent them atrophying altogether. The treatment of such cases demands careful attention, for a person with defective vision of one eye is more than 50 per cent. nearer blindness than he with two good eyes. Not only cannot he see so well, but from that very fact is more liable to accident, especially on the amblyopic side, and, therefore, to sympathetic ophthalmia, all other conditions being equal, which may end in blindness. The refractive errors must be corrected by suitable glasses; the squint should, if existing, be operated on early.

In cases where the amblyopic eye is more hypermetropic than the other, it should be daily exercised by itself, its correcting glass having been ascertained by the shadow test or direct ophthalmoscopy, it should be worn while the sound eye is covered, and used both for distant and near vision, and by perseverance will so improve in many cases as to be able in time to be used with the other eye. Even when that result is not attainable, the amblyopic should be worked up to its best standard, and constantly practised, so that should any mishap befall the other eye, it may become a useful member.

The younger the patient the better is the chance of obtaining a satisfactory result, and it is thus very important that teachers and guardians of the young should be observant, and when they find that the children under their care have difficulty over reading, hold their heads down to their books, are unable to see the black-board, have any tendency to squint, or even complain of headache after reading, often caused by excessive efforts at accommodation, in ametropic eyes, should communicate with the medical or other authority of the school, with a view to having their sight carefully tested. A rough preliminary test might be tried by alternately covering the eyes one at a time, and seeing which eye can see the smaller letter on the black-board from the same distance, and then trying the same with small print at a distance of six to fourteen inches from the child's eye. When the eyes are found to see different sized print and letters, they may feel sure that the eyes should be properly examined, and the errors of refraction being corrected thus early by suitable spectacles, the relapse into amblyopia may be prevented.

